# Effective antioxidant, antimicrobial and anticancer activities of essential oils of horticultural aromatic crops in northern Egypt

**DOI:** 10.1186/s12906-018-2262-1

**Published:** 2018-07-13

**Authors:** Hosam O. Elansary, Samir A. M. Abdelgaleil, Eman A. Mahmoud, Kowiyou Yessoufou, Khalid Elhindi, Salah El-Hendawy

**Affiliations:** 1 Plant production Department, College of Food and Agriculture Sciences, King Saud University, P.O. Box 2460, Riyadh, 11451 Saudi Arabia; 2 Floriculture, Ornamental Horticulture and Garden Design Department, Faculty of Agriculture (El-Shatby), Alexandria University, Alexandria, Egypt; 3 Department of Geography, Environmental Management and Energy Studies, University of Johannesburg, Auckland Park Kingsway Campus (APK) campus, 2006 South Africa; 4 Department of Pesticide Chemistry and Technology, Faculty of Agriculture, Alexandria University, ElShatby, Alexandria, 21545 Egypt; 5 Department of Food Industries, Damietta University, Damietta, Egypt

**Keywords:** Antioxidants, Ornamental plants, Essential oils, Chemical composition

## Abstract

**Background:**

Identifying ornamental plants as new natural antioxidant and antimicrobial sources is always of great importance for the ornamental and horticultural industries.

**Methods:**

The antimicrobial activities of leaves and fruits peel essential oils of twelve ornamental and horticultural crops were determined by screening against wide spectrum of fungi and bacteria, and their respective in vitro antioxidant capacity was evaluated. Furthermore, the anticancer activities against several cancer cells, and one normal human cell line (HEK-293) were examined.

**Results:**

*Origanum vulgare* L. essential oil showed the best antioxidant, antibacterial and anticancer activities compared to screened crops by means of the DPPH and linoleic acid assays for antioxidants, MIC and MBC values for antibacterial activities and IC_50_ for antiproliferative activities. Such important activities in *O. vulgare* was attributed to high pulegone ratio (77.45%) as revealed by the GC/MS assay. *Rosmarinus officinallis* L. essential oil showed the highest antifungal activities by means of lowest MIC and MFC values which might be attributed to 1, 8-cineole (19.60%), camphor (17.01%) and α-pinene (15.12%).

**Conclusion:**

We suggest that oxygenated monoterpenes (i.e. linalool, terpinen-4-ol and pulegone) and monoterpene hydrocarbons play an important role in the essential oil antioxidant, antibacterial, antifungal and anticancer activities of diverse Egyptian ornamental and horticultural crops. Some species showed bioactivities similar to standards compounds and might be suitable for pharmaceutical and food industries.

## Background

In an attempt to preserve human health and avoid food losses in the face of global growing food insecurity, synthetic preservatives were introduced in the food industries [1, 2]. Unfortunately, some of these preservatives were reported to cause undesirable health side effects to humans [1, 2]. For example, lipid peroxidation in stored food may lead to rancidity and reduction of food quality [3–5]. The consumption of spoiled food can cause a wide spectrum of human diseases. Furthermore, the development of resistant microorganisms to synthetic preservatives is another threat facing the continuous use of these chemicals.

Consequently, renewed interest has been placed on the discovery and use of natural bioactive resources in medicinal plants to control diseases, food spoilage microorganisms and oxidation [6–11]. Such medicinal plants include ornamental and horticultural crops that contain essential oils that are bioactive against the development of certain microorganisms [12–15]. The essential oils with high content of phenolics are recognized as strong antioxidants [16–20] and have antimicrobial activities against different types of microorganisms [21–27].

Essential oils might be used as anticancer agents as found before in *Pinus koraiensis* against HCT116 colorectal cancer cells [28], *Myracrodruon urundeuva* against HeLa, HEK-293, and Vero E6 cells [29] and Yemeni medicinal plants against A-427, 5637 and MCF-7 cancer cells [30]. Such activities might be attributed to major essential oil constitutes including cineol, capillin and others [31, 32]. There are dozens of unstudied medicinal horticultural crops in Egypt with certain traditional uses such as skin, face and hair treatments [33] and urological, gastrointestinal, respiratory, neurological, cardiological and immunological diseases control [34]. This great diversity of alternative medicine application of essential oils and leaf extracts might be of pharmaceutical importance that need to be explored.

The study investigated the antioxidant and antimicrobial activities of essential oils obtained from important horticultural and economic ornamental medicinal plants. We also associate the main components of the essential oil with specific antioxidant and antimicrobial properties found to explore novel crop additive values.

## Methods

### Plant materials

The leaves or fruit peels of twelve plant species were collected from Alexandria, Behera and Matrouh in northern Egypt in the spring and summer of 2016. The leaves were obtained from mature non flowering herbal plants of *Rosmarinus officinalis L., Artemisia judaica* L., *A. monosperma*, *Origanum vulgare* L. and *Pelargonium graveolens* L’Her. Also the mature leaves of ornamental non trees of  *Rosmarinus officinalis Callistemon viminalis G.* Don, *Cupressus macrocarpa* Gordon , *Schinus molle* L., and *Thuja occidentalis* L. The fruit peels were obtained from mature fruits of *Citrus aurantifolia* Swingle, *C. limon* (L.) and *C. paradisi*. The plant samples were collected during the months of April to August, 2016. Plants were identified by Prof. Samir Abdelgaleil and Assoc. Prof. Hosam Elansary. Specimens were vouchered  in Department of Pesticide Chemistry and Technology, Alexandria University.

### Essential oils analysis

The leaves were dried for five days (26 ± 1 °C) while the fruit peels were used fresh. The essential oils were obtained by hydrodistillation in a Clevenger-type apparatus for 1 h. Gas chromatography/mass spectrometry (Hewlett Packard 5890) apparatus detected the the essential oil constitutes after dilution in diethyl ether. Gas chromatography column properties and injection conditions matches those described [3]. Oil compositions were identified by comparing retention time and indices of identified constitutes to n-alkanes (C_10_–C_36_, Sigma-Aldrich, Cairo) subjected to the same conditions. NIST Ver. 2.0 and Wiley libraries were used as well for the identificaton of the compounds.

### Microorganisms and cell cultures

The antimicrobial activities were investigated against fungi and bacteria. Fungi included *Aspergillus flavus* (ATCC 9643), *A. ochraceus* (ATCC 12066), *A. niger* (ATCC 6275), *Penicillium ochrochloron* (ATCC 48663), *P. funiculosum* (ATCC 56755), and *Candida albicans* (ATCC 12066)). Bacteria included *Bacillus cereus* (ATCC 14579), *Micrococcus flavus* (ATCC 10240), *Listeria monocytogenes* (clinical isolate), *Staphylococcus aureus* (ATCC 6538), *Pseudomonas aeruginosa* (ATCC 27853), *Dickeya solani* (DS 0432–1) and *Escherichia coli* (ATCC 35210),. The first four bacteria are Gram-positive while the last three are Gram-negative. The microorganisms are either of economic importance for the agricultural industry (e.g. *L. monocytogenes*, *D. solani* and *S. aureus*) or affect human health mainly such as *C. albicans*. Fungi and bacteria were obtained from Alexandria University. Cell cultures including breast adenocarcinoma (MCF-7), cervical adenocarcinoma (HeLa), T-cell lymphoblast (Jurkat), colon adenocarcinoma (HT-29) and urinary bladder carcinoma (T24) were purchased from American Type Culture Collection.

### Antioxidants

The antioxidant activity of the essential oils was estimated using the DPPH [35] and the *β*-carotene-linoleic acid methods [35]. Positive control (BHT) and negative control were treated as samples and a calibration curve was obtained. The amount of the samples that inhibited 50% of each antioxidant solution (IC_50_) were considered as the antioxidant activities and experiments were repeated twice.

### Antifungal activity assay

The antifungal activities of the essential oils were estimated using the microdilution method [36]. The minimum inhibitory concentration (MIC) was the lowest concentration inhibiting the fungal growth using a binocular. The minimum fungicidal concentration (MFC) was estimated by serial sub-cultivations (0.1–4.0 mg/mL). The MFC was determined as the concentration causing no visible growth and killing 99.5% of the original inoculum. Fluconazole (FLZ) and ketoconazole (KLZ) were used as positive control and experiments were repeated twice.

### Antibacterial activity assay

The antibacterial activities were determined by the micro-dilution method [36]. To determine the minimum inhibitory (MIC) and minimum bactericidal (MBC) concentrations the MIC was considered as the lowest concentrations causing no growth using the binocular. The MBC was quantified using serial sub-cultivation of each bacterium (0.1–2.0 mg/mL) into 100 μL of TSB and incubated for one day. MBC was the lowest concentration showed no growth and killed 99.5% of the original inoculum. Experiments were performed twice and negative control (5% DMSO) as well as positive (streptomycin and ampicillin, 0.01–10 mg/mL) were used.

### Antiproliferative activity assay

Essential oils antiproliferative activities against MCF-7, HeLa, Jurkat, HT-29,T24 and HEK-293 followed the MTT method [37] with modifications. Five doses of leaf extracts were used to reach a final concentration of 50, 100, 200, 300, and 400 μg/mL culture media. Negative controls and positive (vinblastine sulfate and taxol) were used.

Furthermore, IC_50_ values were obtained by plotting percentage of cell viability against extract concentration and expressed in μg/ml.

## Results

### Essential oils GC-MS analyses

The essential oils percentage and analyses (Table 1) showed that each species has its own chemical fingerprint. The major oil constitutes (%) were β –thujone (49.83) and chrysanthenone (10.88) in *A. judaica* In *A. monosperma*, capillene (36.86) was the major compound while 1,8-cineole (71.77) was the major in *C. viminalis*. The limonene (40.19) and β-pinene (19.65) were the major compounds in *C. aurantifolia*. In *C*. *lemon*,  limonene (56.30) and *β*-pinene (8.81) were also the major compounds while in *C*. *paradisi* limonene (74.29) and linalool (4.61) were the main compounds. Terpinen-4-ol (20.29) and sabinene (18.67) were the major compounds in *C. macrocarpa*while pulegone (77.45) and menthone (4.86) were majors in *O. vulgare*. β-citronellol (35.92) and geraniol (11.66) were major in *P. graveolens*while 1,8-cineole (19.60), camphor (17.01) and α-pinene (15.12) were major in *R. officinallis*. α-phellandrene (29.87) and β-phellandrene (21.08) were the major compounds in *S. molle*while α-pinene (35.49) and δ-3-carene (25.42) were the major in *T. occidentalis*.

**Table 1:** Major constituents of essential oils extracted from twelve Egyptian plant species

Plant name Oil yield (% F.W., *v*/*w*)	Major components (%, RI^a^)
*Artemisia judaica* (0.2)	*β* –Thujone (49.83,1100), Chrysanthenone (10.88,1125), *α*- Thujone (8.21,1116), 1,8-Cineole (4.91,1034), L-Camphor (3.0,1192), Artemisia alcohol (2.20,1083)
*Artemisia monosperma* (0.8)	Capillene (36.86,1446), capillin (14.68,1572), γ-Terpinene (12.46,1047), *β*-Pinene (7.85,964), *cis*-Ocimene (3.26,1043), Terpinen-4-ol (2.59,1192)
*Callistemon viminalis* (0.5)	1,8-Cineole (71.77,1034), α-Pinene (11.47,946), Terpinen-4-ol (3.18,1192), Octadecanoic acid (3.08,2172), 1-Phellandrene (1.30,1054)
*Citrus aurantifolia* (0.75)	Limonene (40.19,1029), *β*-Pinene (19.65,964), *α*-Citral (8.14,1240), γ-Terpinene (6.34,1047), α-Terpineol (3.71,1185), Terpinen-4-ol (2.62,1192)
*Citrus limon* (0.2)	Limonene (56.30,1029), *β*-Pinene (8.81,964), *γ*-Terpinene (6.42,1047), α-Citral (4.96,1240), β-Citral (3.83,1216), α-Terpineol (3.38,1185)
*Citrus paradisi* (0.12)	Limonene (74.29,1029), Linalool (4.61,1117), Linalool oxide (4.18,1088), β-Citral (2.66,1216), α-Fenchol (1.99,1168), Nootkatone (1.78,1800)
*Cupressus macrocarpa* (0.45)	Terpinen-4-ol (20.29,1192), Sabinene (18.67,974), β-Citronellol (13.01,1225), γ-Terpinene (7.59,1047), Camphor (6.66,1139), α-Terpinene (4.50,1018)
*Origanum vulgare* (0.5)	Pulegone (77.45, 1237), Menthone (4.86, 1152), *cis*-Isopulegone (2.22, 1161), Piperitenone (2.13, 1253), Limonene (1.08, 1029), Myrcene (0.66,984)
*Pelargonium graveolens* (0.09)	β-Citronellol (35.92,1225), Geraniol (11.66,1233), Citronellylformate (11.40,1275), Linalool (9.63,1117), (+)-Isomenthone (6.36,1164), σ-Selinene (5.52,1484)
*Rosmarinus officinalis* (0.33)	1,8-Cineole (19.60,1034), Camphor (17.01,1139), α-Pinene (15.12,946), Verbenone (9.55,1204), Borneol (8.17,1188), Linalool (5.32,1117)
*Schinus molle* (0.88)	α-Phellandrene (29.87,1005), β-Phellandrene (21.08,1031), Elemol (13.00,1547), τ-Muurolol (5.35,1641), γ-Eudesmol (4.48,1629), σ-Cadinene (3.99,1524)
*Thuja occidentalis* (0.25)	α-Pinene (35.49,946), δ-3-Carene (25.42,1004), α-Cedrol (9.05,1585), α-Terpinolene (6.76,1092), Limonene (4.91,1029), Myrcene (2.77,984)

^a^Retention indices related to a homologous series of n-alkanes (C_10_–C_36_, Sigma-Aldrich, Cairo) analyzed in the same conditions and computer matching with the NIST mass spectral search program Ver. 2.0 and Wiley libraries

The results of chemical analysis indicated that components including limonene, *α*-pinene were found in more than one species, while others were species specific. Essential oils major constitutes could be divided into three groups including oxygenated monoterpenes (i.e. linalool and pulegone); monoterpene hydrocarbons (i.e. limonene and sabinene) and sesquiterpene hydrocarbons (i.e., σ-cadinene and σ-selinene).

### Antioxidant activities

The essential oil of *O. vulgare* showed significantly the highest antioxidant activities with IC_50_ values of 2.8 and 1.1 mg/L in the DPPH and linoleic acid assays, respectively compared to other essential oils (Table 2). The oils of *A. judaica*, *A. monosperma* and *C. viminalis* followed *O. vulgare* oil in their antioxidant activities and their IC_50_ values ranged between 4.7–5.3 and 2.7–3.3 mg/L in the DPPH and linoleic acid assays, respectively. The oil of *S. molle* showed the lowest antioxidant activity among all essential oils examined in the study. In addition, the oils of *C. limon, C. macrocarpa, P. graveolens* and *R. officinallis* showed no significant differences regarding their antioxidant activities.

**Table 2:** Antioxidant activity of essential oils using the corresponding concentrations measured by DPPH and *β*-carotene-linoleic acid methods^a^

Essential oil	IC_50_ (mg/L)	
	DPPH radical scavenging	*β*-Carotene-linoleic acid
*Artemisia judaica*	4.7 ± 0.1b	2.7 ± 0.1b
*Artemisia monosperma*	5.3 ± 0.3c^b^	3.3 ± 0.1cd
*Callistemon viminalis*	5.2 ± 0.1c	3.2 ± 0.1c
*Citrus aurantifolia*	7.2 ± 0.3e	5.6 ± 0.3f
*Citrus limon*	6.1 ± 0.3d	4.2 ± 0.1e
*Cupressus macrocarpa*	6.1 ± 0.1d	4.2 ± 0.3e
*Origanum vulgare*	2.8 ± 0.3a	1.1 ± 0.1a
*Pelargonium graveolens*	6.2 ± 0.1d	4.1 ± 0.3e
*Rosmarinus officinalis*	6.0 ± 0.1d	4.2 ± 0.2e
*Schinus molle*	8.9 ± 0.1f	6.6 ± 0.3 g
*Thuja occidentalis*	5.6 ± 0.3 cd	3.6 ± 0.4d
Butylated hydroxytoluene (BHT)	2.9 ± 0.1a	2.6 ± 0.2b

^a^Values are expressed as means ± SD

^b^Means followed by different letters within a column indicate significant differences between treatments based on LSD test (*P* ≤ 0.05)

### Antifungal activities

The activities of the essential oils were expressed as MIC (Table 3) and MFC (Table 4). In general, all examined oils showed antifungal activities against *A. flavus, A. ochraceus, A. niger, P. funiculosum, P. ochrochloron* and *C. albicans*. The MIC and MFC values varied between 0.16 – 1.31 and 0.33–> 4 mg/mL, respectively. The oils of *R. officinallis, O. vulgare, C. macrocarpa* and *C. aurantifolia* showed the lowest MIC values compared to other plants species. The oil of *R. officinallis* showed the highest antifungal activities among studied essential oils by means of lowest values of MIC and MFC against the six fungi. The most resistant fungi were *C. albicans, P. funiculosum* and *A. niger* showing the highest MIC and MFC values. The essential oils of *R. officinallis* and *O. vulgare* revealed comparable activities to commercial reagents.

**Table 3:** Minimum inhibitory concentration (MIC) of essential oils on fungal strains

Essential oil	MIC (mg/mL)					
	*A. flavus*	*A. ochraceus*	*A. niger*	*P. funiculosum*	*P. ochrochloron*	*C. albicans*
*Artemisia judaica*	0.31 ± 0.01	0.38 ± 0.02	0.31 ± 0.01	2.95 ± 0.11	0.41 ± 0.03	0.72 ± 0.03
*Artemisia monosperma*	0.22 ± 0.03	0.29 ± 0.01	0.27 ± 0.03	0.71 ± 0.03	0.33 ± 0.01	0.72 ± 0.11
*Callistemon viminals*	0.25 ± 0.03	0.43 ± 0.01	0.51 ± 0.03	0.96 ± 0.07	0.52 ± 0.05	0.63 ± 0.03
*Citrus aurantifolia*	0.18 ± 0.01	0.19 ± 0.01	0.27 ± 0.01	1.31 ± 0.11	0.41 ± 0.03	0.35 ± 0.01
*Citrus limon*	0.33 ± 0.01	0.29 ± 0.01	0.57 ± 0.03	3.55 ± 0.21	0.57 ± 0.03	0.39 ± 0.03
*Citrus paradisi*	0.38 ± 0.01	0.52 ± 0.03	0.81 ± 0.03	1.35 ± 0.09	0.42 ± 0.01	0.81 ± 0.05
*Cupressus macrocarpa*	0.17 ± 0.01	0.20 ± 0.01	0.26 ± 0.02	0.25 ± 0.02	0.28 ± 0.01	0.72 ± 0.05
*Origanum vulgare*	0.16 ± 0.01	0.18 ± 0.02	0.22 ± 0.01	0.25 ± 0.01	0.33 ± 0.01	0.26 ± 0.01
*Pelargonium graveolens*	0.23 ± 0.01	0.45 ± 0.05	0.39 ± 0.01	0.52 ± 0.01	0.21 ± 0.01	0.83 ± 0.05
*Rosmarinus officinalis*	0.15 ± 0.01	0.27 ± 0.01	0.17 ± 0.01	0.23 ± 0.01	0.21 ± 0.03	0.22 ± 0.01
*Schinus molle*	0.27 ± 0.01	0.31 ± 0.01	0.61 ± 0.05	0.43 ± 0.01	0.47 ± 0.01	0.97 ± 0.09
*Thuja occidentalis*	0.27 ± 0.01	0.41 ± 0.05	0.45 ± 0.03	0.51 ± 0.03	0.19 ± 0.01	1.21 ± 0.07
Fuconazole	0.14 ± 0.01	0.19 ± 0.01	0.16 ± 0.01	0.11 ± 0.01	0.20 ± 0.01	0.11 ± 0.01
Ketoconazole	0.21 ± 0.01	0.20 ± 0.01	0.12 ± 0.01	2.00 ± 0.09	0.20 ± 0.01	0.21 ± 0.01

**Table 4:** Minimum fungicidal concentration (MFC) of essential oils on fungal strains

Essential oil	MFC (mg/mL)					
	*A. flavus*	*A. ochraceus*	*A. niger*	*P. funiculosum*	*P. ochrochloron*	*Candida albicans*
*Artemisia judaica*	0.77 ± 0.03	0.63 ± 0.03	0.85 ± 0.03	> 4	0.95 ± 0.05	1.86 ± 0.09
*Artemisia monosperma*	0.46 ± 0.01	0.61 ± 0.02	0.87 ± 0.05	1.81 ± 0.10	0.61 ± 0.03	1.63 ± 0.13
*Callistemon viminalis*	0.63 ± 0.05	0.95 ± 0.05	1.13 ± 0.08	> 2	0.95 ± 0.03	1.1 ± 0.05
*Citrus aurantifolia*	0.39 ± 0.03	0.41 ± 0.02	0.56 ± 0.02	> 2	0.83 ± 0.03	0.71 ± 0.03
*Citrus limon*	0.74 ± 0.05	0.76 ± 0.04	1.43 ± 0.07	> 4	1.41 ± 0.08	0.91 ± 0.05
*Citrus paradisi*	0.76 ± 0.03	> 2	> 2	3.91 ± 0.17	0.93 ± 0.05	1.59 ± 0.07
*Cupressus macrocarpa*	0.37 ± 0.03	0.48 ± 0.03	0.49 ± 0.03	0.73 ± 0.03	0.68 ± 0.03	1.53 ± 0.15
*Origanum vulgare*	0.35 ± 0.01	0.47 ± 0.03	0.47 ± 0.03	0.61 ± 0.01	0.71 ± 0.02	0.63 ± 0.03
*Pelargonium graveolens*	0.47 ± 0.01	1.21 ± 0.09	0.83 ± 0.01	1.19 ± 0.08	0.49 ± 0.03	1.92 ± 0.10
*Rosmarinus officinalis*	0.33 ± 0.01	0.59 ± 0.03	0.38 ± 0.03	0.57 ± 0.03	0.47 ± 0.03	0.49 ± 0.05
*Schinus molle*	0.67 ± 0.07	0.67 ± 0.07	1.38 ± 0.09	1.12 ± 0.06	0.89 ± 0.05	2.3 ± 0.11
*Thuja occidentalis*	0.63 ± 0.03	0.96 ± 0.12	1.75 ± 0.11	1.11 ± 0.09	0.52 ± 0.02	2.51 ± 0.11
Fuconazole	0.20 ± 0.03	0.32 ± 0.01	0.27 ± 0.01	0.24 ± 0.01	0.31 ± 0.01	0.20 ± 0.01
Ketoconazole	0.39 ± 0.03	0.40 ± 0.01	0.19 ± 0.01	3.75 ± 0.07	0.40 ± 0.03	0.41 ± 0.03

### The antibacterial activities

There were large differences regarding essential oils antibacterial activities by means of MIC (Table 5) and MBC (Table 6). The oils of *O. vulgare*, *C. macrocarpa* and *C. paradisi* showed the highest antibacterial activities with MIC values ranged between 0.11–0.76 mg/mL against examined bacteria. *O. vulgare*, *C. macrocarpa* and *C. paradise* were the three most active essential oils, the MBC was in the range of 0.21 to 1.59. The most resistant bacterium in this case of *D. solani* and *L. monocytogenes*. In general, the highest MIC values were recorded for the essential oils of *A. monosperma, C. lemon*, *R. officinallis* and *S. molle*. Most essential oils showed slightly higher MIC values compared to antibiotics, however, *O. vulgare* showed comparable values to antibiotics in some cases. The antibacterial activities were higher than antibiotics.

**Table 5:** Minimum inhibitory concentrations (MIC) of essential oils on bacterial strains

Essential oil	MIC (mg/mL)						
	*B. cereus*	*D. solani*	*E. coli*	*L. monocytogenes*	*M. flavus*	*P. aeruginosa*	*S. aureus*
*Artemisia judaica*	0.35 ± 0.01	0.39 ± 0.01	0.45 ± 0.01	0.31 ± 0.01	0.23 ± 0.03	0.16 ± 0.01	0.27 ± 0.01
*Artemisia monosperma*	0.43 ± 0.01	0.40 ± 0.01	0.58 ± 0.03	0.52 ± 0.01	0.31 ± 0.01	0.52 ± 0.01	0.38 ± 0.00
*Callistemon viminals*	0.37 ± 0.01	0.31 ± 0.01	0.49 ± 0.01	0.33 ± 0.01	0.31 ± 0.03	0.39 ± 0.01	0.41 ± 0.01
*Citrus aurantifolia*	0.23 ± 0.01	0.49 ± 0.01	0.21 ± 0.01	0.31 ± 0.01	0.27 ± 0.03	0.21 ± 0.01	0.25 ± 0.01
*Citrus limon*	0.53 ± 0.01	0.49 ± 0.01	0.27 ± 0.01	0.43 ± 0.01	0.83 ± 0.07	0.21 ± 0.01	0.43 ± 0.01
*Citrus paradisi*	0.15 ± 0.01	0.27 ± 0.01	0.21 ± 0.01	0.20 ± 0.01	0.13 ± 0.03	0.15 ± 0.01	0.17 ± 0.01
*Cupressus macrocarpa*	0.19 ± 0.01	0.29 ± 0.01	0.26 ± 0.01	0.21 ± 0.01	0.13 ± 0.03	0.19 ± 0.01	0.24 ± 0.01
*Origanum vulgare*	0.11 ± 0.01	0.76 ± 0.01	0.25 ± 0.01	0.40 ± 0.01	0.21 ± 0.03	0.15 ± 0.01	0.28 ± 0.01
*Pelargonium graveolens*	0.25 ± 0.01	0.27 ± 0.01	0.19 ± 0.01	0.25 ± 0.02	0.15 ± 0.01	0.21 ± 0.01	0.20 ± 0.01
*Rosmarinus officinalis*	0.40 ± 0.01	0.89 ± 0.01	0.43 ± 0.01	0.52 ± 0.01	0.25 ± 0.03	0.22 ± 0.01	0.69 ± 0.01
*Schinus molle*	0.29 ± 0.01	0.63 ± 0.01	0.72 ± 0.01	0.71 ± 0.03	0.42 ± 0.03	0.51 ± 0.01	0.37 ± 0.01
*Thuja occidentalis*	0.33 ± 0.03	0.41 ± 0.03	0.26 ± 0.01	0.31 ± 0.01	0.20 ± 0.01	0.41 ± 0.01	0.25 ± 0.01
Streptomycin	0.07 ± 0.01	0.21 ± 0.01	0.09 ± 0.01	0.18 ± 0.01	0.9 ± 0.01	0.07 ± 0.01	0.21 ± 0.01
Ampicillin	0.10 ± 0.01	0.30 ± 0.01	0.24 ± 0.01	0.18 ± 0.01	0.11 ± 0.01	0.12 ± 0.01	0.11 ± 0.01

**Table 6:** Minimum bactericidal concentration (MBC) of essential oils on bacterial strains

Essential oil	MBC (mg/mL)						
	*B. cereus*	*D. solani*	*E. coli*	*L. monocytogenes*	*M. flavus*	*P. aeruginosa*	*S. aureus*
*Artemisia judaica*	0.63 ± 0.03	1.13 ± 0.01	0.93 ± 0.05	0.72 ± 0.01	0.53 ± 0.03	0.31 ± 0.03	0.53 ± 0.03
*Artemisia monosperma*	0.89 ± 0.03	0.84 ± 0.05	1.31 ± 0.09	1.23 ± 0.09	0.71 ± 0.03	1.96 ± 0.01	0.65 ± 0.03
*Callistemon viminals*	0.67 ± 0.01	0.75 ± 0.01	0.91 ± 0.07	0.73 ± 0.05	0.76 ± 0.01	1.13 ± 0.09	0.96 ± 0.03
*Citrus aurantifolia*	0.49 ± 0.03	0.81 ± 0.03	0.47 ± 0.03	0.63 ± 0.07	0.94 ± 0.01	0.61 ± 0.01	0.53 ± 0.00
*Citrus limon*	1.31 ± 0.01	0.81 ± 0.01	0.49 ± 0.03	0.93 ± 0.07	> 2	0.40 ± 0.03	0.93 ± 0.05
*Citrus paradisi*	0.37 ± 0.01	0.61 ± 0.01	0.43 ± 0.03	0.83 ± 0.03	0.25 ± 0.01	0.31 ± 0.01	0.63 ± 0.03
*Cupressus macrocarpa*	0.38 ± 0.01	0.63 ± 0.01	0.51 ± 0.03	0.57 ± 0.03	0.27 ± 0.01	0.53 ± 0.03	0.47 ± 0.03
*Origanum vulgare*	0.21 ± 0.01	1.59 ± 0.01	0.58 ± 0.03	0.83 ± 0.03	0.42 ± 0.01	0.34 ± 0.03	0.67 ± 0.03
*Pelargonium graveolens*	0.59 ± 0.03	0.67 ± 0.03	0.83 ± 0.03	0.46 ± 0.03	0.33 ± 0.01	0.43 ± 0.03	0.51 ± 0.03
*Rosmarinus officinalis*	0.91 ± 0.01	1.81 ± 0.01	0.82 ± 0.03	1.15 ± 0.07	0.71 ± 0.01	0.93 ± 0.01	1.73 ± 0.03
*Schinus molle*	0.83 ± 0.01	1.57 ± 0.01	1.78 ± 0.03	> 2	0.98 ± 0.01	1.13 ± 0.01	0.89 ± 0.03
*Thuja occidentalis*	0.79 ± 0.05	0.83 ± 0.01	0.71 ± 0.03	0.97 ± 0.05	0.53 ± 0.01	0.94 ± 0.01	0.53 ± 0.03
Streptomycin	0.14 ± 0.01	0.40 ± 0.02	0.40 ± 0.01	0.35 ± 0.01	0.19 ± 0.01	0.15 ± 0.01	0.41 ± 0.03
Ampicillin	0.18 ± 0.01	0.55 ± 0.01	0.41 ± 0.03	0.30 ± 0.01	0.18 ± 0.01	0.25 ± 0.01	0.19 ± 0.03

### Anticancer activities

There were variations in essential oils anticancer activities against selected cancer cell lines as shown in Table 7. The inhibition (expressed as IC_50_) of different types of cancer cells proliferation ranged between 8.1 and ˃300 μg/ml. The highest antiproliferation activities were found in *O. vulgare*, *Citrus* sp. and *A. monosperma* against MCF-7, HeLa, Jurkat, HT-29 and T24 cancer cells. The lowest antiproliferative activities were found in the essential oils of *P. graveolens*. No inhibition activity was found in any oil against HEK-293 (kidney epithelial).

**Table 7:** In vitro antiproliferative activity [IC_50_ (μg/ml)] of twelve aromatic plants essential oils on cancer cell lines

	MCF-7	HeLa	Jurkat	HT-29	T24	HEK-293
*Artemisia judaica*	28.51 ± 1.1	54.13 ± 1.5	63.71 ± 1.6	73.01 ± 2.1	171.13 ± 1.8	>300
*Artemisia monosperma*	15.15 ± 1.1	9.1 ± 0.1	11.0 ± 0.5	10.1 ± 0.5	119.0 ± 2.5	>300
*Callistemon viminals*	25.15 ± 0.3	18.75 ± 1.5	53.10 ± 3.5	10.51 ± 1.0	166.15 ± 2.8	>300
*Citrus aurantifolia*	11.11 ± 0.3	58.75 ± 1.5	17.10 ± 1.5	230.84 ± 4.1	>300	>300
*Citrus limon*	9.52 ± 1.6	51.04 ± 1.2	15.34 ± 1.2	231.91 ± 5.1	216.7 ± 4.1	>300
*Citrus paradisi*	8.1 ± 1.5	46.15 ± 1.8	14.52 ± 1.9	220 ± 5.3	113.6 ± 5.1	>400
*Cupressus macrocarpa*	25.4 ± 2.6	24.16 ± 1.6	30.54 ± 3.4	124.8 ± 5.2	>300	>300
*Origanum vulgare*	8.11 ± 1.0	13.41 ± 1.1	27.05 ± 2.1	12.18 ± 1.4	105.5 ± 2.3	>300
*Pelargonium graveolens*	61.0 ± 1.5	51.24 ± 3.1	178.5 ± 2.8	195.33 ± 5.4	270.13 ± 7.1	>300
*Rosmarinus officinalis*	36.5 ± 2.1	27.25 ± 1.5	73.11 ± 2.9	18.17 ± 2.0	118.31 ± 2.8	>300
*Schinus molle*	41.33 ± 2.1	119.5 ± 2.6	14.85 ± 1.7	18.35 ± 1.3	>300	>300
*Thuja occidentalis*	57.35 ± 2.3	22.5 ± 1.7	95.52 ± 1.3	125.5 ± 3.9	>300	>300
Vinblastine sulfate	–	2.5 ± 0.08	0.1 ± 0.05	21.4 ± 1.5	63.31 ± 1.7	51.5 ± 2.1
Taxol	0.08 ± 0.005	–	–	–	–	–

**Table 8:** Common names and edible parts of twelve ornamental and horticultural Egyptian plant species

Plant name	Common name	Edible/economic parts	Common uses
*Artemisia judaica*	Judean wormwood	Leaves and flowers	Spice, soft drink and cosmetic uses [37]
*Artemisia monosperma*	Delile	Leaves and flowers	Spice, soft drink and cosmetic uses [37]
*Callistemon viminalis*	weeping bottlebrush	Leaves and flowers	Ornamental and source of antioxidants, antifungal and antibacterial products [47]
*Citrus aurantifolia*	Key lime	Fruits and leaves	The fruits are eaten and the essential oils are extracted from the fruit coat. The leaves are used for medicinal puposes [46, 58]
*Citrus limon*	lemon	Fruits and leaves	The fruits are eaten and the essential oils are extracted from the fruit coat. The leaves are used for medicinal puposes [46, 58]
*Citrus paradisi*	grapefruit	Fruits and leaves	The fruits are eaten and the essential oils are extracted from the fruit coat. The leaves are used for medicinal puposes [46, 58]
*Cupressus macrocarpa*	Monterey cypress	Stem, leaves and cones	Ornamental and source of antioxidants, antifungal and antibacterial products [38]
*Origanum vulgare*	wild marjoram	Leaves and flowers	Spice, soft drink and cosmetic uses [25]
*Pelargonium graveolens*	Geranium	Leaves and flowers	Spice, soft drink and cosmetic uses [45]
*Rosmarinus officinalis*	Rosemary	Leaves and flowers	Spice, soft drink and cosmetic uses [25]
*Schinus molle*	American pepper	Leaves, flowers and fruits	Ornamental and source of antioxidants, antifungal and antibacterial products [48]
*Thuja occidentalis*	northern white-cedar	Stem, leaves and cones	Ornamental and source of antioxidants, antifungal and antibacterial products [49]

## Discussion

Essential oils showed high variation in term of composition even among closely related species (i.e. species of the same genera), which is in agreement with previous reports on several plants [39–41]. All essential oils examined showed antioxidant capacities; however, *O. vulgare* showed the highest antioxidant activities. This might be attributed to specific essential oil such as the pulegone (77.45%). The pulegone is a known compound found in the family Lamiaceae and has been associated with high antioxidant activities [42–44]. Although previous studies reported that pulegone is the major oil component of *O. vulgare* [44], environmental conditions of the plant may alter the chemical composition of the oil [41, 43] leading to the variation of chemical composition that we found among closely related species. Similarly, many other species showed high antioxidant activities (e.g. *A. judaica, A. monosperma* and *C. viminalis*). cajor components and their synergetic effects. β α. Specifically, we found a high ratio of β –thujone (49.83%) and *α*-thujone (8.21%) in *A. Judaica*, supporting the previous report in *Artemisia sieberi* Besser in Italy and Iran [45]. The essential oil ratio found in Italy and Iran [45] is slightly higher than that found in our study. However, *A. monosperma* showed high ratios of α-pinene and terpinen-4-ol which are known to have noticeable antioxidant activities [46]. The high antioxidant activities that we found in relation with the presence of *β*–thujone may indicate that this compound may play a major role in the antioxidant activities of different *Artemisia* species.

The essential oil in *R. officinallis* showed the highest antifungal activities by means of lowest MIC and MFC values which might be attributed to 1,8-cineole (19.60%), camphor (17.01%) and α-pinene (15.12%) as the major oil constituents as previously reported [47]. It was reported that 1,8-cineole, terpinen-4-ol and other compounds showed strong in-vitro antifungal activities against some plant pathogenic fungi including *Fusarium cerealis, Aspergillus tubingensis* and *A. carbonarius* [48]. In the current study, we report strong antifungal activities of the 1,8-cineole-rich *R. officinallis* against plant and human pathogenic fungi including *A. flavus, A. ochraceus, A. niger, P. funiculosum, P. ochrochloron* and *C. albicans*. The oil of *O. vulgare* followed *R. officinallis* in term of level of antifungal activities, and such interesting bioactivities of the essential oil might be attributed to the presence of pulegone (77.45%) and the menthone (4.86%).

The essential oil in *C. macrocarpa* showed high antifungal activities compared to other species, which is attributed to the presence of essential antifungal compounds including terpinen-4-ol (20.29%), sabinene (18.67%), γ-terpinene (7.59%) and α-terpinene (4.50%) in agreement with previous investigations on other plants [46]. High ratios of limonene (40.19%) and β-pinene (19.65%) were found in *C. aurantifolia*, confirming previous report [40]. 

In the current study, we document the highest antibacterial activity for the *O. vulgare* essential oil by means of MIC and MBC values compared to other species, potentially because of the high pulegone ratio. Previous investigations reported strong antibacterial activities of essential oils of the pulegone-rich *Mentha longifolia* L. and pulegone-rich *O. vulgare* against different bacteria such as *Pantoea agglumerans* and human pathogenic *Acinetobacter baumannii*, respectively [43, 44]. The high antifungal as well as antibacterial activities of pulegone rich essential oils suggest that this compound might have a wide spectrum of antifungal and antibacterial activities. The oils of *C. macrocarpa* and *C. paradise* followed *O. vulgare* in their level of antibacterial activities, which might be attributed to terpinen-4-ol and sabinene in *C. macrocarpa* as well as limonene and linalool in *C. paradise*. A strong antibacterial activity of five different terpenoids was reported [49] against *Campylobacter* spp. In addition, it was previously reported that the monoterpene terpinen-4-ol enhanced the antibacterial performance of *Melaleuca alternifolia* essential oils against *S. aureus* and *E. coli* [50]. In the same trend, limonene is the major component of citrus essential oils in most species and was proven to have specific bacterial inactivation mechanism in *E. coli* and others [51] thus justifying the noticeable antibacterial activities of *C. paradisi*. However, other species studied here (*C. limon* and *C. aurantifolia*) showed slightly lower antibacterial activities which could be partially explained by lower ratios of limonene and synergetic effects with other oil constituents such as the linalool in *C. paradisi*. Some essential oils in *R. officinallis* and *O. vulgare* showed comparable antibacterial and antifungal activities to antibiotics in some cases, which may indicate that these essential oils might be useful sources of natural products for commercial applications in the pharmaceutical and food industries.

Several studies indicated that Gram-positive bacteria are less resistant than negative ones against natural products such as essential oils [52]. This resistance had been attributed to the additional outer membrane (this is likely the case in our study) and that most resistant bacterium was *D. solani*. Finally, the essential oils compositions and percentages obtained from this study are comparable to studies on geranium [53], citrus [54], *C. viminalis* [55], *R. officinallis* [47], *Artimisia* [45], and *O. vulgare* [44]. However, the essential oils composition of the current collection of horticultural crops differed in some cases from those reported in the literature for *S. molle* (mainly composed of pinene [56], *T. occidentalis* (mainly composed of thujone and fenchone [57] and *O. vulgare* (mainly composed of pulegone). Interestingly, other report on *S. molle* found that the essential oil might be composed of cubenol (27.1%) and caryophyllene oxide (15.3%) [58]. Indeed, a great diversity is known in essential oil plants due to environmental as well as genetic factors.

Few species showed promising anticancer activities against the proliferation of cancer cells. The main effect of the essential oils is attributed to main constitutes such as the capillin in *A. monosperama*, limonene in the *Citrus* sp. and pulegone in *O. vulgare*. In agreement with our results in *Artemisia judaica* oil (mainly composed of thujone), Torres et al. [59] reported that the thujone enriched fraction has potential anticancer activities. The use of capillin (1–10 uM), isolated from *A. monosperama*, *inhibited* cell proliferation of HT29, MIA PaCa-2 and HEp-2 [32]. Several recent studies investigated the anticancer activities of several compounds. Murata et al. [31] found that 1, 8-cineole (the main component of *Callistemon viminals*) suppress the prolifieration of colon cancer cells by inducing apoptosis (see Miller et al. [60] for in-depth review). On one hand, Shapira et al. [61] found that Terpinen-4-ol inhibited different cancer cells and Shehab and Abu-Gharbieh, [62] suggested that the essential oil of *Micromeria Fruticosa* L. (58.5% pulegone) has antitumor activities against Human Colon Cancer and MCF7 with IC_50_ at 10 and 12.7 μg/Ml, respectively. Fayed [63] on the other hand reported that geranium essential oil (29.90% citronellol) had the highest anticancer activity with the LC_50_ values of 62.50 μg/ml in NB4 cell line and 86.5 μg/ml in HL-60 cell line whereas Lin et al. [64] reported that α-Phellandrene found in *Schinus molle* influenced cell cycle and apoptosis in murine leukemia WEHI-3 cells. Chen et al. [65, 66] found that α-pinene has antitumor activities through inducing cell cycle arrest.

We aimed to highlight the biological activities in ornamental and horticultural aromatic plants against pathogenic microbes in the Mediterranean region. We demonstrated their antioxidant activities by two different methods and their promising role as natural antioxidant resources. One of the important factors influencing the decision of the farmer to grow specific horticultural crop is the availability of different ways of marketing such as the use of the whole crop as a fresh crop or the use of the essential oil for the pharmaceutical industries (Table 8). Here the bioactivity that we found for these crops is an added value that may encourage farmers to grow these crops and increase the marketing possibilities of their end crops. On the medical level, the pharmaceutical values of those medical crops are promising for pathogenic diseases and human cancer control.

## Conclusion

*O. vulgare*had the best antioxidant and antibacterial activities with a high and unique pulegone ratio (77.45%). The essential oil of *R. officinallis* essential oil showed the highest antifungal activities by means of lowest MIC and MFC values which might be attributed to 1,8-cineole, camphor and α-pinene. The essential oils of *O. vulgare*, *Citrus* sp. and *A. monosperma* showed the highest antiproliferation activities against different cancer cells. Oxygenated monoterpenes (i.e. linalool and pulegone) as well as monoterpene hydrocarbons including pinenes plays a pivotal role in the antioxidant, antibacterial, antifungal and anticancer activities of the essential oils of different horticultural aromatic plants. Some species showed antioxidant and antimicrobial activities comparable to standard compounds, which may indicate that these crops are valuable resources of natural compounds useful for pharmaceutical and food industries.
